# ICD-1/BTF3 antagonizes SKN-1-mediated endoderm specification in *Caenorhabditis elegans*

**DOI:** 10.17912/micropub.biology.000167

**Published:** 2019-10-04

**Authors:** Chee Kiang Ewe, Yamila N Torres Cleuren, Geneva Alok, Joel H Rothman

**Affiliations:** 1 Department of MCD Biology and Neuroscience Research Institute, University of California Santa Barbara, CA, USA; 2 School of Biological Sciences, University of Auckland, Auckland, New Zealand

**Figure 1:**
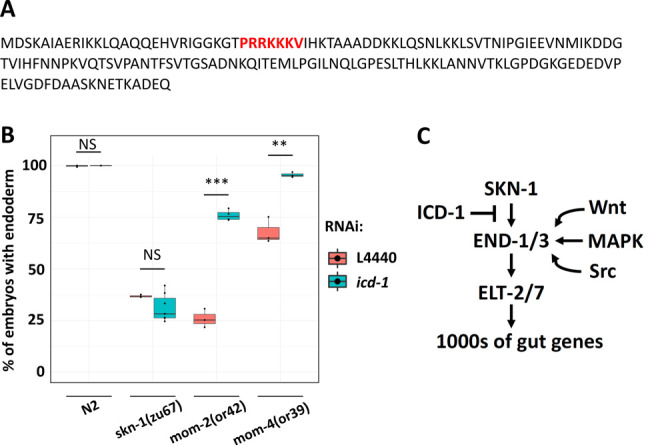
A) Amino acid sequence of ICD-1. The putative nuclear localization signal is highlighted in red. B) The effects of *icd-1* RNAi on N2, *skn-1(zu67),* *mom-2(or42)*, and *mom-4(or39)* absence-of-endoderm phenotype. At least three replicates were performed per experiment with >200 embryos scored per experiment. Student t-test (NS p-value> 0.05, ** p-value ≤ 0.01, *** p-value ≤ 0.001). C) Hypothesized model of ICD-1 function in endoderm specification, positing that it antagonizes the SKN-1 input upstream of END-1/3.

## Description

The entire *C. elegans* intestine is derived from a single endodermal progenitor cell (E), the posterior daughter arising from the asymmetric division of the EMS blastomere. During early embryonic development, maternally provided SKN-1/Nrf2 activates the mesendoderm gene regulatory network (GRN) in both E and its sister, MS. A triply redundant Wnt/MAPK/Src signaling system from the neighboring P_2_ blastomere polarizes EMS, resulting in activation of E fate on the side contacting it. In MS, and in an unsignaled E cell, POP-1/Tcf represses expression of the redundant endoderm specifying factors, the END-1 and -3 GATA-type transcription factors. In a normal E cell, Wnt (initiated by the MOM-2/Wnt ligand) and MAPK signaling (through the MOM-4 MAPKKK) converge on POP-1 to convert it from a repressor to an activator of the *end* genes which, in collaboration with SKN-1, activates E cell fate (Thorpe *et al.* 1997; Maduro and Rothman 2002; McGhee 2007; Maduro 2017).

Basal transcription factor 3 (BTF3) facilitates transfer of nascent polypeptide chains into mitochondria and regulates transcription in plants and animals (Jamil *et al.* 2015). We previously found that the *C. elegans* BTF-3 orthologue, ICD-1, is required to prevent apoptosis: eliminating *icd-1* leads to increased cell death in embryos and larvae (Bloss *et al.* 2003). However, consistent with its potential function as a transcription factor, ICD-1 contains a putative nuclear localization signal in the N-terminus (Fig.1A) (Lange *et al.* 2007). Here, we report that ICD-1 performs a function in endoderm specification. We found that *icd-1* RNAi does not affect endoderm specification in a wild-type N2 background or the gut-less phenotype of *skn-1(-)* embryos. However, knockdown of *icd-1* strongly suppresses the absence of gut in *mom-2/Wnt(-)* embryos (*mom-2(or42)*: 26.0% ± s.d. 4.5% with gut *vs. mom-2(or42); icd-1(RNAi)*: 75.9% ± s.d. 2.6%). Similarly, depleting ICD-1 rescues the gut-less phenotype of *mom-4/Tak1(-)* embryos (*mom-4(or39)*: 67.9% ± s.d. 6.3% with gut *vs. mom-4(or39); icd-1(RNAi)*: 95.5% ± s.d. 1.2%) (Fig. 1B). Our findings suggest a model in which ICD-1 antagonizes the SKN-1 input, perhaps by preventing SKN-1 from binding to *end-1/3* promotors. This possibility is also consistent with the finding that competition between ICD-1 and SKN-1 is seen in the context of the unfolded protein response (UPR). SKN-1 binds to and activates *hsp-4*, which codes for an endoplasmic reticulum chaperone BiP (Glover-Cutter *et al.* 2013). Depleting ICD-1 results in upregulation of *hsp-4* and activation of the UPR (Arsenovic *et al.* 2012), suggesting that ICD-1 and SKN-1 perform opposing functions in other contexts. In this hypothesized model, ICD-1 may act to fine-tune developmental signals, thereby ensuring proper specification and differentiation of endoderm (Fig. 1C).

## Reagents

Strains

JJ185 *dpy-13(e184) skn-1(zu67) IV; mDp1 (IV;f)*

JR3936 *dpy-13(e184) skn-1(zu67) IV/nT1 [qIs51] (IV;V)*

EU384 *dpy-11(e1180) mom-2(or42) V/nT1 [let-?(m435)] (IV;V).*

EU414 *unc-13(e1091) mom-4(or39)/hT2 I; +/hT2 [bli-4(e937) let-?(h661)] III.*

RNAi and quantification of endoderm specification

*E. coli* HT115 expressing *icd-1* dsRNA was obtained from the Ahringer RNAi library (Kamath *et al.* 2003). RNAi experiments for embryonic endoderm specification were performed as described (Torres Cleuren *et al.* 2019). In brief, bacteria were grown at 37^o^C in LB containing 50 μg/ml ampicillin. The overnight culture was then diluted 1:10. After 4 hours of incubation at 37^o^C, 1 mM of IPTG was added and 60 μl was seeded onto 35 mm agar plates containing 1 mM IPTG and 25 μg/ml carbenicillin. Seeded plates were allowed to dry overnight before use. 20-30 L4 or young adults were placed on the seeded RNAi plate. 24 hours later, they were transferred to a fresh RNAi plate and allowed to lay eggs for four hours. The adults were then removed, leaving the embryos to develop for an extra 5-7 hours. Embryos expressing birefringent gut granules were quantified and imaged on an agar pad using a Nikon Ti-E inverted microscope under dark field with polarized light (Clokey and Jacobson 1986; Hermann *et al.* 2005). All experiments were performed at 20^o^C.
